# NurA Is Endowed with Endo- and Exonuclease Activities that Are Modulated by HerA: New Insight into Their Role in DNA-End Processing

**DOI:** 10.1371/journal.pone.0142345

**Published:** 2015-11-11

**Authors:** Mariarosaria De Falco, Federico Catalano, Mosè Rossi, Maria Ciaramella, Mariarita De Felice

**Affiliations:** Institute of Biosciences and Bioresources, Consiglio Nazionale delle Ricerche, Naples, 80131, Italy; Institute of Molecular Genetics IMG-CNR, ITALY

## Abstract

The nuclease NurA and the ATPase HerA are present in all known thermophilic archaea and cooperate with the highly conserved MRE11/RAD50 proteins to facilitate efficient DNA double-strand break end processing during homologous recombinational repair. However, contradictory results have been reported on the exact activities and mutual dependence of these two enzymes. To understand the functional relationship between these two enzymes we deeply characterized *Sulfolobus solfataricus* NurA and HerA proteins. We found that NurA is endowed with exo- and endonuclease activities on various DNA substrates, including linear (single-stranded and double stranded) as well as circular molecules (single stranded and supercoiled double-stranded). All these activities are not strictly dependent on the presence of HerA, require divalent ions (preferably Mn^2+^), and are inhibited by the presence of ATP. The endo- and exonculease activities have distinct requirements: whereas the exonuclease activity on linear DNA fragments is stimulated by HerA and depends on the catalytic D58 residue, the endonuclease activity on circular double-stranded DNA is HerA-independent and is not affected by the D58A mutation. On the basis of our results we propose a mechanism of action of NurA/HerA complex during DNA end processing.

## Introduction

In all organisms genomic DNA is continuously subjected to a wide variety of lesions; the rapid detection of the damage and the subsequent accurate repair is crucial to maintain genomic integrity. DNA lesions are generated either by external agents, such as UV light, mechanical stress, ionizing radiation, carcinogens, or intrinsic errors occurring during DNA replication, recombination and aberrant chromosome segregation. Among the various types of DNA lesions, double-strand breaks (DSBs) are one of the most harmful because, if not correctly repaired, may result in chromosome loss or deletions, translocations, and genomic instability causing a profound influence in proliferation of normal cells and eventually cell death. In eukaryotic cells, two major DSB repair pathways are known: Non-Homologous End Joining (NHEJ) and Homologous Recombination (HR). The NHEJ pathway is an error-prone process in which the two ends of the broken chromosome are ligated back together directly. HR is one of the most important DSB repair pathways [1,2] and, in contrast to NHEJ, it is a high-fidelity mechanism since it relies upon homologous DNA sequences and generates error-free repaired products. During HR initiation, enzymatic resection of DNA ends generates 3′-single-stranded DNA (ssDNA) overhangs that are necessary for loading recombinases (RecA/Rad51/RadA) [3,4].

HR has been investigated extensively in bacteria and eukarya. It has been suggested that in eukaryotes, DSBs that occur in the G1 phase of the cell cycle are most likely to be repaired *via* NHEJ, while those occurring in the S/G2 phase are preferentially processed *via* HR [5,6]. The HR machinery is comprised of a core protein complex containing Mre11-Rad50-Nbs1 (human, MRN) or Mre11-Rad50-Xrs2 (*Saccharomyces cerevisiae*, *MRX*); this complex, in conjunction with Ctp1/CtIP (or Sae2), recognizes DSBs and removes few nucleotides to form early intermediates during HR [7–13]. EXO1/Exo1 nuclease, BLM/Sgs1 helicase, and DNA2/Dna2 nuclease bind to these intermediates and generate 3′-ssDNA overhangs [1,9,11–13] that are then utilized in Rad51-dependent strand exchange [9,11,14–16].

Archaea appear to primarily encode the HR pathway for DSB repair, since homologous of several eukaryotic HR components have been identified, including Mre11 and Rad50 and the recombinase RadA [17,18]. A few studies were reported on the functional role of these putative HR proteins in archaea [19–23]. The archaeal Mre11-Rad50 complex senses and processes DSB ends through 3′- to 5′- exonuclease and endonuclease activities to form short 3′-overhangs [24,25]. Two archaeal genes, *herA* and *nurA*, usually located in operons, have been implicated in HR because of their conserved genomic association with *mre11* and *rad50* [26], moreover, the four genes are co-induced in response to UV irradiation [27,28].Several data support the hypothesis that these four proteins are involved in HR DNA end resection [25,29,30]. *HerA and NurA* products are believed to be the functional homologous of eukaryotic Exo1/EXO1, Dna2/DNA2, and Sgs1/BLM proteins since the corresponding genes have not been found in archaea so far. This hypothesis has been supported by biochemical characterization of the encoded proteins: HerA proteins characterized from a few archaeal species all exhibit ATPase activity and for some of them (*Sulfolobus acidocaldarius and S*. *todokaii*) also dipolar helicase activity, while, so far, NurA has been considered a 5′→3′ exonuclease and ssDNA endonuclease [31–35]. Several studies demonstrated that Mre11, Rad50, HerA and NurA are capable of working in concert to process dsDNA *in vitro* [25,29,30,35,36]. However, contradictory results have been reported on the properties of this complex: NurA from *S*. *acidocaldarius* was reported to display both single-stranded endonuclease and 5′→3′ exonuclease activity on single-stranded and double-stranded DNA [32]; in contrast the very similar NurA from *S*. *solfataricus* was found to be completely inactive in the absence of HerA [30]; moreover, NurA from *Pyrococcus furiosus* was reported to have a weak Mn^2+^ dependent 5′ to 3′ exonuclease activity, but no nicking activity [25,36], as also reported for the *Deinococcus radiodurans* protein [37]. Recently, *in vivo* studies demonstrated that all four genes of the operon are essential for *S*. *islandicus* viability, and in particular the ATPase activtiy of HerA, the nuclease activity of NurA and their interaction [38].

In this study, we used a biochemical approach in order to clarify the properties and functional interaction of NurA and HerA from the hyperthermophilic archaeon *S*. *solfataricus*. We show that purified NurA is endowed with exo- and endonuclease activities on various DNA substrates; these activities are inhibited by ATP and restored in the presence of HerA. HerA modulates NurA nuclease activity on different substrates, but is not able to stimulate NurA endonuclease activity on circular double-stranded DNA. Moreover, the endonuclease activity of NurA on circular double-stranded DNA was not abolished by mutation of the catalytic D58 residue, which is essential for NurA nuclease activity on linear substrates. On the basis of our results we propose a mechanism of action of NurA/HerA complex during DNA end processing.

## Material and Methods

### Construction of Plasmids for Protein Over-Expression in *Escherichia coli*

The open reading frame (ORF) encoding the SSO2248 (NurA) was amplified by PCR from the P2 strain genomic DNA of *Sulfolobus solfataricus*, using the High Fidelity PCR system (Roche Diagnostics) with the synthetic oligonucleotides designed to insert NdeI and BamHI restriction sites at 5’ and 3’ ends, respectively. The sequence of the primers was as follows: NurA-NdeI-for: 5’-GGTTCATATGATAAGAAAAATATATGATAAGTTAGTAGAA-3’; NurA-BamHI-rev: 5’-GGTTGGATCCTCAAGTCTCCTCCCTCACAAAACTTTTATT-3’ (the restriction sites sequence are underlined). The amplified fragment was cloned into NdeI/BamHI-digested pet29a vector (Novagen) to create the construct named NurApet29a.

The open reading frame (ORF) encoding the SSO2251 (HerA) was amplified as above using the synthetic oligonucleotides designed to insert BamHI and EcoRI restriction sites at 5’ and 3’ ends, respectively. The sequence of the primers was as follows: HerA-BamHI-for: 5’-GGTTGGATCCATGATAATTGGTTATGTAATTGGTCAAGCT-3’; HerA-EcoRI-rev: 5’-GGTTTGAATTCTCAATCACCAATTTCTGTTCCAAAGTCAGC-3’ (the restriction sites sequence are underlined). The amplified fragment was cloned into BamHI /EcoRI -digested pet29a vector (Novagen) to create the construct named HerApet29a.

The D58A mutant NurA was generated from the wild-type clone by site-directed mutagenesis using the synthetic oligonucleotides designed to substitute an Aspartic Acid with an Alanine in position 58 of the aminoacid sequence. The oligonucleotide used were: NurAD58Afor 5’-5’- ACTTGTAAATTTGTAGCTATTGCCGGTGGATCTTTTGGTAGACC -3’ NurAD58Arev 5’- 5’- CCACCGGCAATAGCTACAAATTTACAAGTTTCGCTCTGTTCATGG– 3’ (the point-mutation sites are underlined).

All the cloned PCR products were sequenced at the PrimmBiotech DNA Sequencing Service.

### Expression and Purification of Recombinant Proteins

NurA, HerA and NurA D58A were expressed and purified following the same procedure as indicated below.

The E.coli BL21-CodonPlus(DE3)-RIL cells (Novagen), transformed with the plasmid of interest were grown at 37°C in 500 ml of LB (Luria–Bertani) medium containing 30 μg/ml chloramphenicol and 30 μg/ml kanamycin. When the culture reached an A600nm of 0.8 OD, protein expression was induced by addition of IPTG 0.2 mM. The bacterial culture was then incubated overnight at 37°C. The cells were harvested by centrifugation and the pellet was resuspended in 20 ml of Buffer A (25 mM Tris-HCl pH 8, 2.5 mM MgCl2, 100 mM NaCl), supplemented with protease inhibitors (Complete Mini EDTA-free Roche). The cells were broken by three consecutive passages through a French pressure cell apparatus (Aminco Co., Silver Spring, MD) at 1500 p.s.i. The resulting lysate was centrifuged for 20 min at 30000 r.p.m. (Beckman rotor 50.2 Ti) at 10°C. The supernatant was heat-treated at 70°C for 10 minutes and centrifuged for 20 min at 30000 r.p.m. (Beckman rotor 50.2 Ti) at 10°C. Soluble fraction was subsequently filtered through a 0.22 μm filter (Millipore) and loaded onto a Mono Q HR 10/100 column (GE Healthcare Life Sciences), connected to an AKTA system (Amersham Biosciences). The elution of proteins was performed with a 30 ml linear gradient of NaCl (from 0.1 M to 1 M, flow rate: 0.5 ml/min). The fractions containing the protein were pooled and the pool was dialyzed against Buffer B (50 mM Tris/HCl pH 8, 5 mM MgCl2, 100 mM NaCl, 10% glycerol). This sample was then loaded onto an affinity Hi-Trap Heparin column (GE Healthcare). 30 ml linear gradient of NaCl (from 0.1 M to 1 M, flow rate: 0.5 ml/min) was applied to the column. The fractions containing the protein were pooled and concentrated on a YM10 ultrafiltration membrane (Amicon).

### Substrate Preparation

The substrates were generated annealing the oligoncleotides reported in Table 1 as described below.

**Table 1:** Oligonucleotides used for DNA substrates preparation.

*Name*	*Sequence*
**ODN1**	5’-GCCGTGATCACCAATGCAGATTGACGAACCTTTGCCCACGT-3’Cy3
**ODN2**	5’ Cy5-GACGTGGGCAAAGGTTCGTCAATGGACTGACAGCTGCATGG-3’
**70leadCy5**	5’ Cy5-CGTGACTTGATGTTAACCCTAACCCTAAGAATTCGGCTTAAGTGAGTGTGAGGATATCATGTACGATAGC-3’
**70 lag**	5’-GCTATCGTACATGATATCCTCACACTCTGAATAGCCGAATTCTTAGGGTTAGGGTTAACATCAAGTCACG-3’
**70 lag bubble**	5’-GCTATCGTACATGATATCCTCACACTTTTTTTTTTTTTTTTTTTTGGGTTAGGGTTAACATCAAGTCACG-3’
**70 lag fork**	5’-TTTTTTTTTTTTTTTTTTTTCACACTCACTTAAGCCGAATTCTTAGGGTTAGGGTTAACATCAAGTCACG-3’
**70 lag emi**	5’-CACACTCACTTAAGCCGAATTCTTAGGGTTAGGGTTAACATCAAGTCACG-3’
**ODN2 comp**	5’-GCCGTGATCACCAATGCAGATTGACGAACCTTTGCCCACGT-3’
**21mer5’Cy5**	5’ Cy5-GCTATCGTACATGATATCCTC-3’
**21mer3'TAM**	5’-GCTATCGTACATGATATCCTC-3’TAM
**17mer5'TAM**	5’TAM-GTTTTCCCAGTCACGAC-3’
**17mer3'TAM**	5’-GTTTTCCCAGTCACGAC-3’TAM

70mer ds was obtained annealing 70 leadCy5 with 70 lag; 70mer EMI fork annealing 70 leadCy5 with 70 lag emi; 70mer fork annealing 70 leadCy5 with 70 lag fork; 70mer bubble annealing 70 leadCy5 with 70 lag bubble; 40mer ds annealing ODN2 with ODN2 comp.

### Nuclease Assay

The catalytic activity of NurA nuclease was assayed on a variety of DNA substrates. Reactions were performed in 10 μl reaction volume, containing 20 mM HEPES/NaOH pH 7.5, 10 mM β-mercaptoehtanol, 30 mM NaCl, 5 mM MnCl_2_ and incubated for 30 minutes at 70°C in a heated-top PCR machine to prevent evaporation.

For supercoiled and linear plasmid the reactions were stopped by the addition of 0.5% SDS, 40 mM EDTA, 0.5 mg/ml proteinase K, 20% glycerol and the products were separated on a 1% agarose 1 X TBE, stained with ethidium bromide and visualized under UV light.

For ds- and ss-DNA fluorescent oligonucleotides reactions were terminated by the addition of 5 μl of stop solution (10 mM EDTA, 97% formammide) and separated on 15% polyacrilammide gel and 8 M urea in 1 X TBE. Gels were visualized by VersaDoc^TM^ MP 4000 system (Bio-Rad).

### DNA Band-Shift Assays

For each substrate, reactions were performed in a final volume of 10 μl containing 2 pmoles of fluorescent DNA in 20 mM HEPES/NaOH (pH 7.5), 3.5 mM β-mercaptoethanol, 50 mM NaCl, 5 mM MgCl_2_ and 2 mM EDTA and the indicated amounts of the different proteins. Following incubation for 10 min at room temperature, 1 μl of 100% glycerol was added and the complexes were separated by electrophoresis through 5% polyacrylamide/bis gels in 0.5 X TBE. To analyze the functional interaction between NurA and HerA, the two proteins were incubated for 20 minutes at 70°C then the DNA was added and the reactions were analyzed after 30 minutes of incubation at room temperature. The products were visualized by VersaDocTM MP 1000 system and quantified using ImageQuant as processing program. The values obtained were plotted against the amount of proteins used for each experiment.

### DNA Helicase Assay

Reaction mixtures (20 μl) containing 2 pmol of fluorescent substrate and the indicated amounts of proteins in helicase assay buffer (25 mM HEPES/NaOH, pH 7.5, 2.5 mM 2-mercaptoethanol, 50 mM sodium acetate, 5 mM ATP, 7.5 mM MgCl_2_) were incubated for 30 min at 60°C in a heated-top PCR machine to prevent evaporation. Reactions were stopped by addition of 5 μl of 5 X Stop Solution (0.5% SDS, 40 mM EDTA, 0.5 mg/ml proteinase K, 20% glycerol, 0.1% bromophenol blue) and the products separated on a 8% polyacrylamide gel in TBE, containing 0.1% SDS at a constant voltage of 150 V. After the electrophoresis the gel was analyzed by VersaDoc^TM^ MP 4000 system.

## Results

### A Nuclease Activity Co-Migrates with NurA Purification Profile

**Fig 1:**
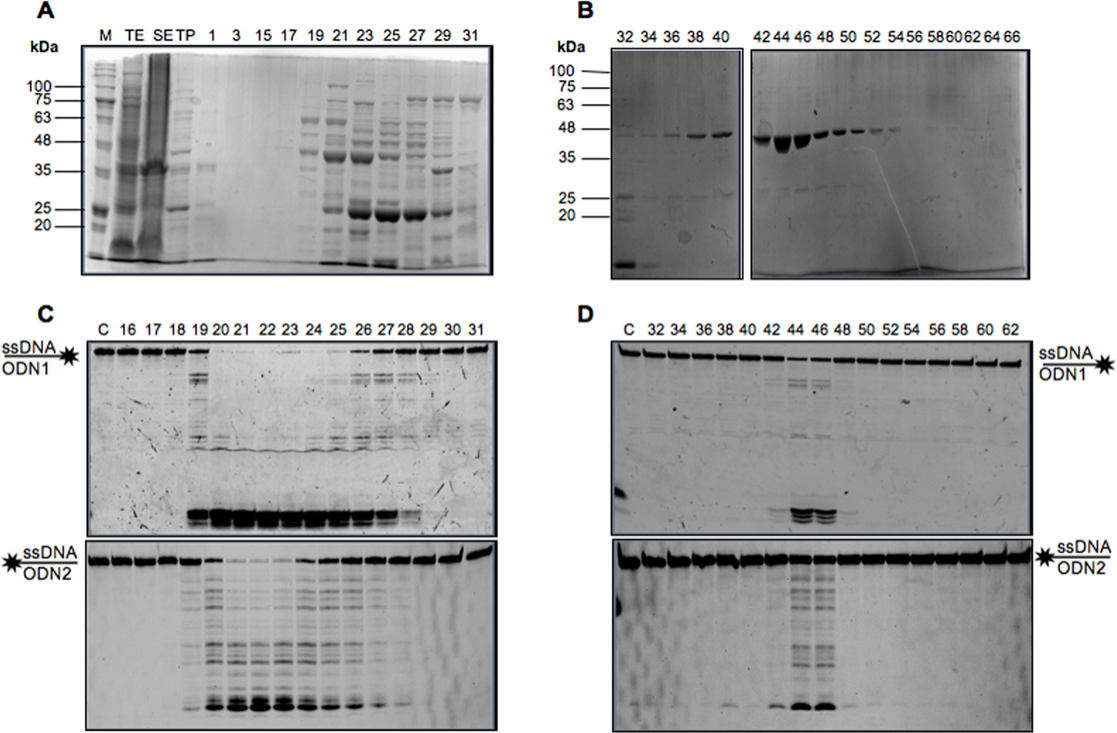
Analysis of *S*. *solfataricus* NurA purification and activity profile. (A) Anion exchange chromatography analysis using a MonoQ column. 20 μl of NurA elution fractions were loaded on SDS-PAGE, on the top of the gel are reported the elution fraction numbers. Markers (M), total extract (TE), soluble extract (SE), thermo-precipitated fraction (TP). (B) Affinity chromatography elution profile using a Heparin column. 20 μl of each fraction, whose number is reported on the top of the gel, were loaded on SDS-PAGE. Nuclease activity profile of NurA eluted from MonoQ column first (C) and Heparin column (D) after. 7 μl of each fraction, whose number is reported on the top of the gel, were tested for their nuclease activity using both ODN1 (top) or ODN2 (bottom) substrates as described in Material and Methods. Lane C is a control lane in which no protein was added in the mixture assay.

When we first expressed recombinant *Sulfolobus solfataricus* NurA in *E*. *coli* we detected a prominent nuclease activity. Since Blackwood JK *et al*. [30] reported that the same protein does not show any nuclease activity on its own, we decided to unequivocally demonstrate that the activity observed was related to NurA. To this aim, we undertook the purification of recombinant NurA and followed the nuclease activity through multiple chromatographic steps. Aliquots of fractions from MonoQ (Fig 1A) and heparin (Fig 1B) columns were incubated with the 40 base oligonucleotides ODN1, fluorescently labelled at its 3’-end with Cy3, or ODN2, labelled at its 5’-end with Cy5 (Table 1). As shown in Fig 1C and 1D, the protein and the nuclease activity peaks were perfectly superimposable (compare panel A and C for Mono Q or B and D for Heparin fractions).

*S*. *solfataricus* NurA and HerA were homogeneously purified from E. *coli* cultures (S1 Fig) and analyzed for their oligomeric states using a Superdex 200 10/300 gel filtration column and they turned out to be dimer (NurA) and hexamer (HerA) in solution. When the two proteins were previously mixed and then loaded onto the same column, they co-eluted earlier than the two proteins run separately, indicating that they form a stable complex (S2 Fig), in agreement with what previously observed by Blackwood JK *et al*. [30]. Moreover these fractions were analyzed for their nuclease activity and both NurA peaks and NurA-HerA peaks were superimposable with the nuclease activity (data not shown). Assays at different temperatures showed that the nuclease activity is thermophilic, with a peak of activity at 70°C (data not shown).

**Fig 2:**
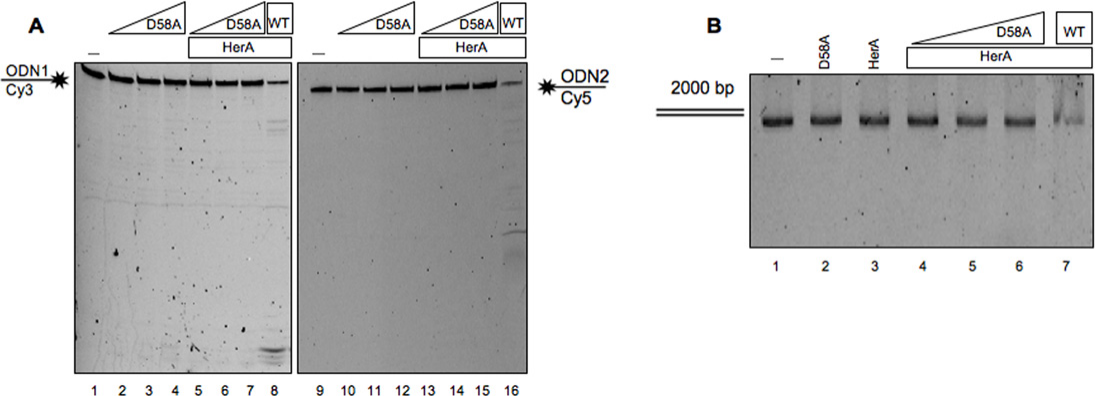
NurA D58A mutant activity assays. (A) Increasing amounts (2.5, 5 and 10 pmoles) of NurA D58A were analyzed for their nuclease activity, on single-stranded DNA, separately (lanes 2–4 and 10–12) or in presence of 12 pmoles of HerA (lanes 5–7 and 13–15) on two different substrates ODN1 and ODN2. Lanes 1 and 9 refer to controls in which the only substrate has been loaded, while lanes 8 and 16 refer to positive controls in which 12 pmoles of NurAwt were incubated in presence of 12 pmoles of HerA. (B) Increasing amounts (2, 6 and 10 pmoles) of NurA D58A were analyzed for their nuclease activity, on linear double-stranded DNA, in presence of 12 pmoles of HerA (lanes 4–6); lane 1 refers to control in which the only substrate has been loaded, lane 2 refers to NurA D58A alone (10 pmoles) and lane 3 refers to HerA alone (12 pmoles).

In order to further demonstrate that the nuclease activity was due to NurA we constructed the D58A mutant in which the aspartic residue, located in NurA catalytic site, was substituted with an alanine [30]. The mutant was expressed and purified exactly as previously done for wild-type NurA (see Material and Methods; S3 Fig). As expected, the D58A protein did not show any nuclease activity on linear single-stranded DNA substrates (Fig 2A lanes 2–4 and 10–12) even in the presence of HerA (Fig 2A lanes 5–7 and 13–15). The same experiment was performed on a linear double-stranded DNA (2000 bp). Also in these conditions we did not observe any degradation either in the absence (Fig 2B lane 2) or in the presence (Fig 2B lanes 4–6) of HerA.

### NurA Nuclease Activity Is Inhibited by ATP

**Fig 3:**
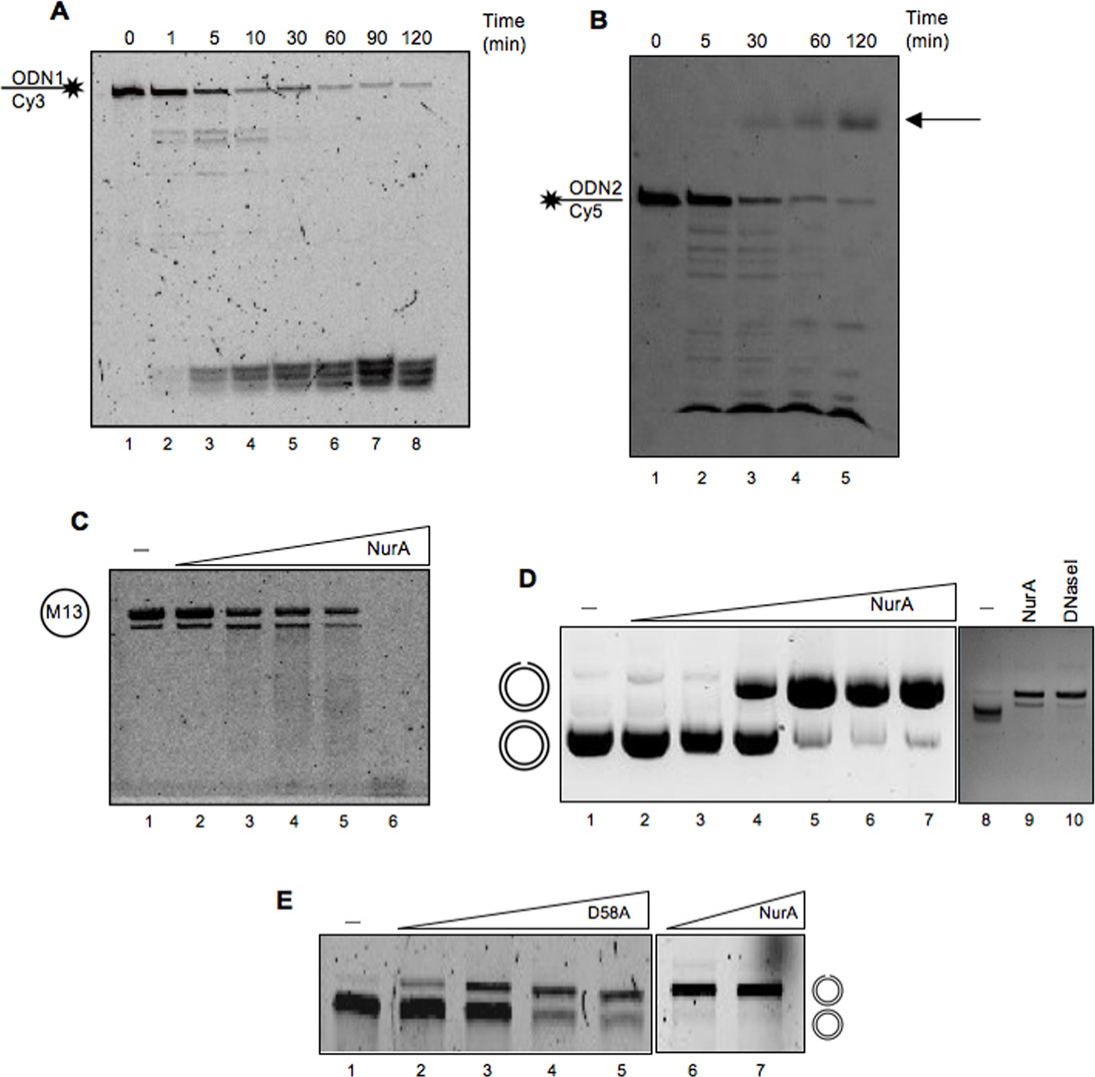
Nuclease activity of NurA on different DNA substrates. NurA nuclease activity on linear single-stranded DNA labeled at its 3’-end (A) or 5’-end (B). A time course analysis has been conducted to analyze NurA nuclease activity on these substrates in order to visualize the whole different products coming out from its activity. (C) NurA endonuclease activity on a single-stranded DNA (M13). Increasing concentrations of NurA (from 0.6 to 12.5 pmoles) have been used to test its endonuclease activity on a circular single-stranded DNA (lanes 2–6). Lane 1 is a control in which M13 has been incubated with no protein. (D) Analysis of NurA endonuclease activity on a circular double-stranded DNA. Increasing amounts of NurA (from 0.1 to 7.5 pmoles) have been analyzed for its endonuclease activity (lanes 2–7). A control lane with no protein has been run (lane 1). Control experiments were conducted to compare the activity of NurA (lane 9) and 0,1 nmol of commercial DNaseI (lane 10). A control lane with no protein has been run (lane 7). (E) Increasing amounts (1, 2.5, 5 and 10 pmoles) of NurA D58A were analyzed for their nuclease activity (lanes 2–5), on circular double-stranded DNA, lane 1 refers to control in which the only substrate has been loaded, lanes 6 and 7 refer to NurA wt alone as positive control (5 and 10 pmoles).

We decided to further characterize NurA nuclease activity. A time course analysis with the ODN1 and ODN2 oligonucleotides showed that NurA degradation activity produced different molecular weight products (Fig 3A and 3B). Cy5 confers an anomalous migration to the oligomers since, when the products are 6 or less nucleotides in length, they start migrating as products with a higher molecular weight, as previously demonstrated by Henneke *et al*. [39], This behavior explains the signal that can be observed in Fig 3B (as indicated by the arrow) which corresponds to a fragment of about 2 nucleotides. This activity might be due to either exo- or endonuclease activity, or both. To test if NurA has endonuclease activity, we used a circular single-stranded DNA (ssM13): increasing amounts of NurA digested the substrate up to completeness (Fig 3C, lane 6). Moreover, when a circular double-stranded DNA was used, NurA promoted the formation of a nicked DNA form, suggesting that NurA possesses single strand DNA endonuclease activity also on double-stranded DNA (nicking activity; Fig 3D). Interestingly, the nicking activity was also observed with the NurA D58A mutant (Fig 3E), suggesting that the ss endonuclease activity is due to a catalytic site distinct from that responsible for the exonuclease observed with linear substrates.

**Fig 4:**
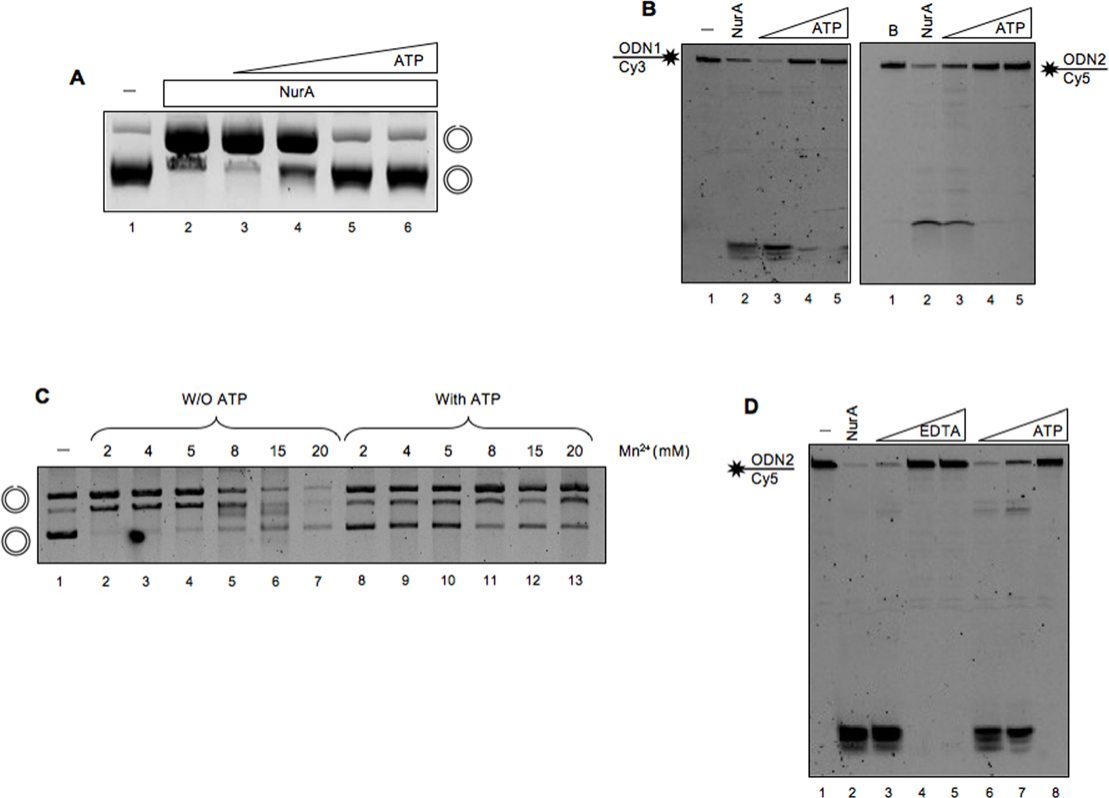
Effect of ATP on NurA nuclease activity. (A) NurA activity has been tested on a circular double-stranded DNA in the absence (lane 2) or in the presence (lane 3–6) of increasing concentrations of ATP (1.5 3, 5, 7 mM). A sample in which no protein was added in the mixture has been loaded and used as control (lane 1). (B) Analysis of ATP effect on NurA nuclease on linear single-stranded substrates. NurA nuclease activity on ODN1 (left) or ODN2 (right) was tested in the absence (lane 2) or in the presence (lanes 3–5) of increasing concentrations of ATP (from 2.5 to 10 mM). Lane 1 refers to a control in which no protein was added in the sample assay. (C) ATP seizes Mn^2+^ ions from the reaction. NurA endonuclease activity has been analyzed with increasing amounts of Mn^2+^ ions in the absence (lanes 2–7) or in the presence (lanes 8–13) of ATP (5 mM). The concentration of Mn^2+^ used is reported on the top of the gel. (D) Effect of EDTA on NurA nuclease activity. NurA nuclease activity on ODN2 has been analyzed, using 9 pmoles of NurA, in the presence of increasing amounts of EDTA or ATP as control (both from 2.5 to 10 mM), lanes 3–5 and 6–8, respectively. A control in which no protein was present was loaded on lane 1, while lane 2 refers to NurA nuclease activity in the absence of neither ATP nor EDTA.

The results reported so far, showing that purified NurA behaves as an exo/endonuclease, are in agreement with those reported by Constantinesco et al. [32], but not with what observed by Blackwood et al. [30]. To investigate the reasons for this discrepancy, we tested NurA nuclease activity under the same assay conditions described by Blackwood et al. [30], and, surprisingly, we found no activity. Since the only substantial difference between the two reaction conditions was the presence of ATP in the latter, we decided to analyze the effect of ATP on NurA nuclease activity. As we can clearly observe in Fig 4A, ATP (5 mM) completely inhibited NurA endonuclease activity. The same experiment was performed also on a linear double- (data not shown) or single-stranded DNA (Fig 4B) and the results obtained indicated a complete inhibition of NurA nuclease activity due to the presence of ATP.

As previously reported [32,36], NurA nuclease activity requires divalent ions; in particular Mn^2+^ was strongly preferred to Mg^2+^ (S4 Fig). We thus hypothesized that ATP might inhibit the nuclease activity by subtracting free Mn^2+^/Mg^2+^ ions from the reaction. Indeed, ATP inhibition was partially overcome by increasing Mn^2+^ ions concentration: in the presence of 6 mM ATP, Mn^2+^concentration higher than 8 mM stimulated NurA nuclease activity, although less efficiently as compared with the reaction with no ATP (Fig 4C). Moreover, addition of EDTA had the same inhibitory effect as ATP (Fig 4D). Taken together these results confirm our hypothesis that ATP subtracts divalent ions from the reaction preventing NurA activity.

### Functional Interaction of NurA and HerA

**Fig 5:**
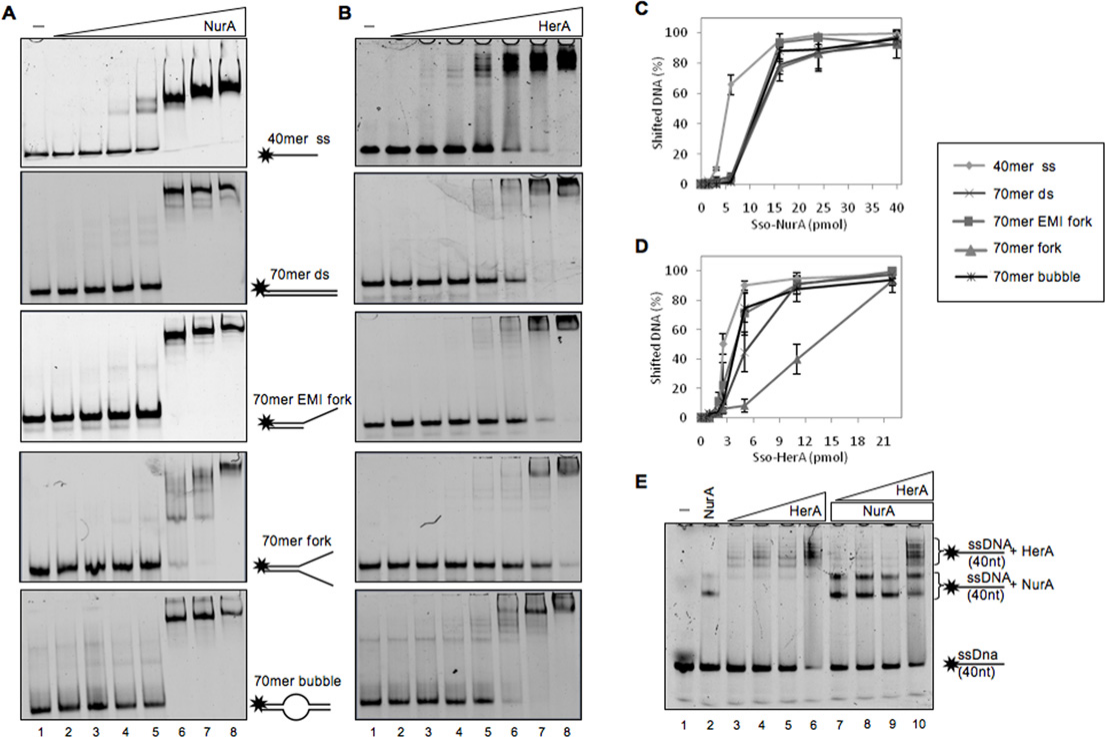
NurA and HerA binding to different DNA structures. Increasing amounts of NurA (A) or HerA (B) have been analyzed for their DNA binding affinity for different DNA structures as described in Material and Methods (single-stranded ODN2, double-stranded, emi-forked, forked and bubble from top to bottom). Substrates with no protein were run on lane 1. The gels have been quantified (as reported in Material and Methods) and the values have been reported on a graph (C and D). The values obtained are the average of three different experiments. (E) HerA promotes NurA DNA binding. DNA binding affinity of NurA/HerA complex on a single-stranded DNA has been analyzed using a fixed amount of NurA (3 pmoles) and increasing amounts of HerA (from 2 to 6 pmoles) separately (lanes 3–6) or together (lanes 7–10).

We decided to undertake an analysis of the binding efficiency of NurA and HerA to various DNA molecules (Table 1, Material and Methods). Whereas NurA showed a slight preference for ssDNA, HerA preferred single stranded, bubble and emiforked DNA substrate with respect to the other DNA molecules tested. When increasing amounts of HerA (from 2 to 6 pmoles) were mixed with 3 pmoles of NurA, we observed a slight increased efficiency of DNA binding by NurA at the expense of HerA (Fig 5E compare lanes 3–6 with 7–10).

**Fig 6:**
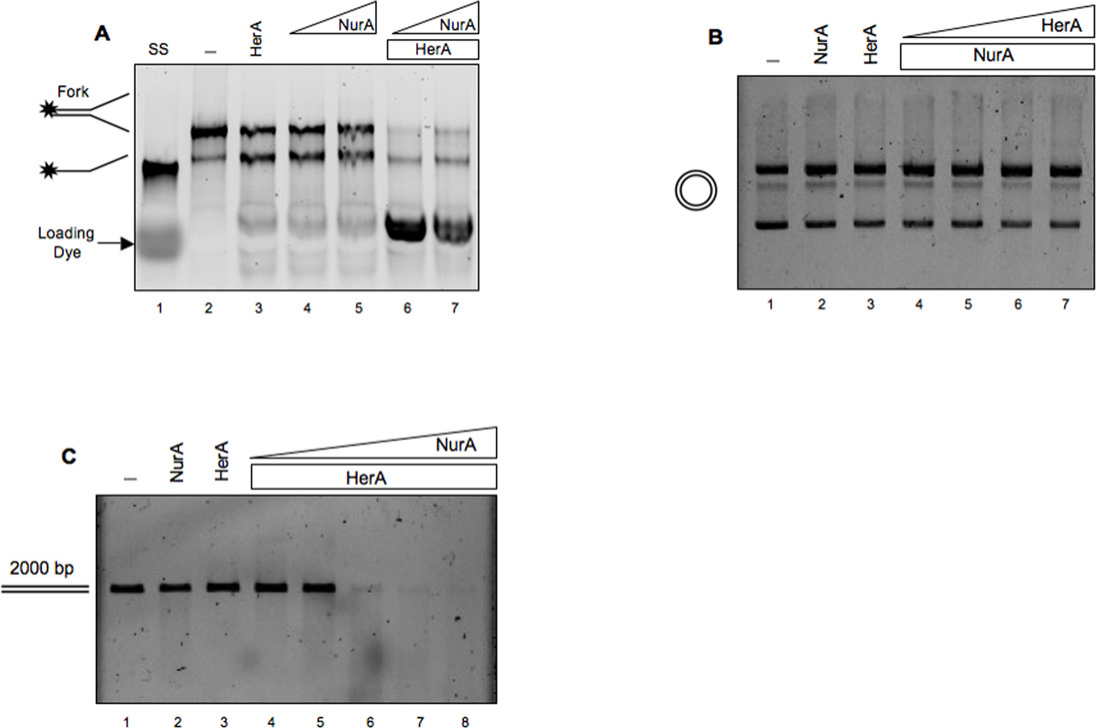
Analysis of NurA/HerA complex activity. (A) HerA DNA helicase activity has been tested on a forked DNA using 12 pmoles of HerA in the absence (lane 3) or in the presence of two different concentrations of NurA, 11 and 20 pmoles (lanes 6–7). NurA was also analyzed without HerA (lanes 4–5) as control. The single-stranded DNA (lane 1) was loaded to visualize its migration, while the forked substrate has been loaded on lane 2. (B) The endonuclease activity of NurA/HerA complex was tested on a circular double-stranded DNA. The two proteins were analyzed separately using 18 pmoles of NurA or 18 pmoles of HerA (lanes 2 and 3, respectively) or together using increasing concentration of HerA from 0.8 to 18 pmoles (lanes 4–7). A control lane in which no protein was added was loaded on lane 1. (C) Nuclease activity of NurA/HerA complex on a linear double-stranded DNA (2000 bp). The two proteins were analyzed separately using 4.7 pmoles of NurA or 3 pmoles of HerA (lanes 2 and 3, respectively) or together using increasing amounts of NurA from 0.4 to 4.7 pmoles and a fixed concentration of HerA (3 pmoles) (lanes 4–8). A control lane in which no protein was added was loaded on lane 1.

Previously, a number of HerA proteins from different species have been tested for helicase activity, with contradictory results. Indeed, HerA from *Methanobacter thermoautotrophicus* was reported to have no activity [33], whereas the protein from *S*. *acidocaldarius* showed weak activity [31]. We thus decided to test our HerA preparation using a forked 70-nucleotides DNA molecule as substrate in a buffer containing magnesium and ATP (see Material and Methods). In agreement with results reported by Blackwood *et al*. [30], we did not observe any helicase activity when HerA was incubated separately (Fig 6A, lane 3); moreover, no activity was observed by NurA alone, since the reaction contains magnesium ions and 5 mM ATP, a condition that does not allow NurA nuclease activity (Fig 6A lanes 4–5). When increasing amounts of NurA were incubated with a fixed amount of HerA, a digestion product was observed (Fig 6A lanes 6–7) suggesting that HerA stimulates NurA nuclease activity; moreover, stimulation of nuclease activity by HerA was observed also in the presence of Mg^2+^ (S5 Fig).

To investigate the effect of HerA on NurA endonuclease activity, we incubated a circular double-stranded DNA with increasing amounts of HerA and a fixed concentration of NurA. As expected, NurA alone did not show any endonuclease activity under these conditions (Fig 6B, lane 2); however, in this case, addition of HerA, even at high concentration, was not able to restore NurA endonuclease activity (Fig 6A, lanes 4–7). Therefore, we decided to perform the same experiment in the absence of ATP using a concentration of NurA at which the endonuclease activity was barely detectable, in order to appreciate even weak stimulation by HerA. Again, no effect of HerA on NurA endonuclease activity was seen (data not shown). In contrast, when we used a linear double-stranded DNA (about 2000 nucleotides) as substrate, HerA was able to restore NurA nuclease activity (Fig 6C, lanes 4–8).

Taken together, these results suggest that NurA nuclease activity is inhibited by ATP and requires the presence of divalent ions; HerA was able to overcome this inhibition on linear, but not circular DNA molecules, suggesting that HerA stimulation of NurA nuclease activity needs free DNA ends. An attractive hypothesis is that HerA possesses a poor helicase activity that is sufficient to induce local unwinding of DNA, and the exposed single strand regions are target for NurA nuclease activity. In alternative, it is also possible that HerA-induced ATP binding/hydrolysis relieves inhibition of NurA nuclease activity by reducing the concentration of free nucleotide.

### HerA Modulates the Pattern of NurA Nuclease Activity

**Fig 7:**
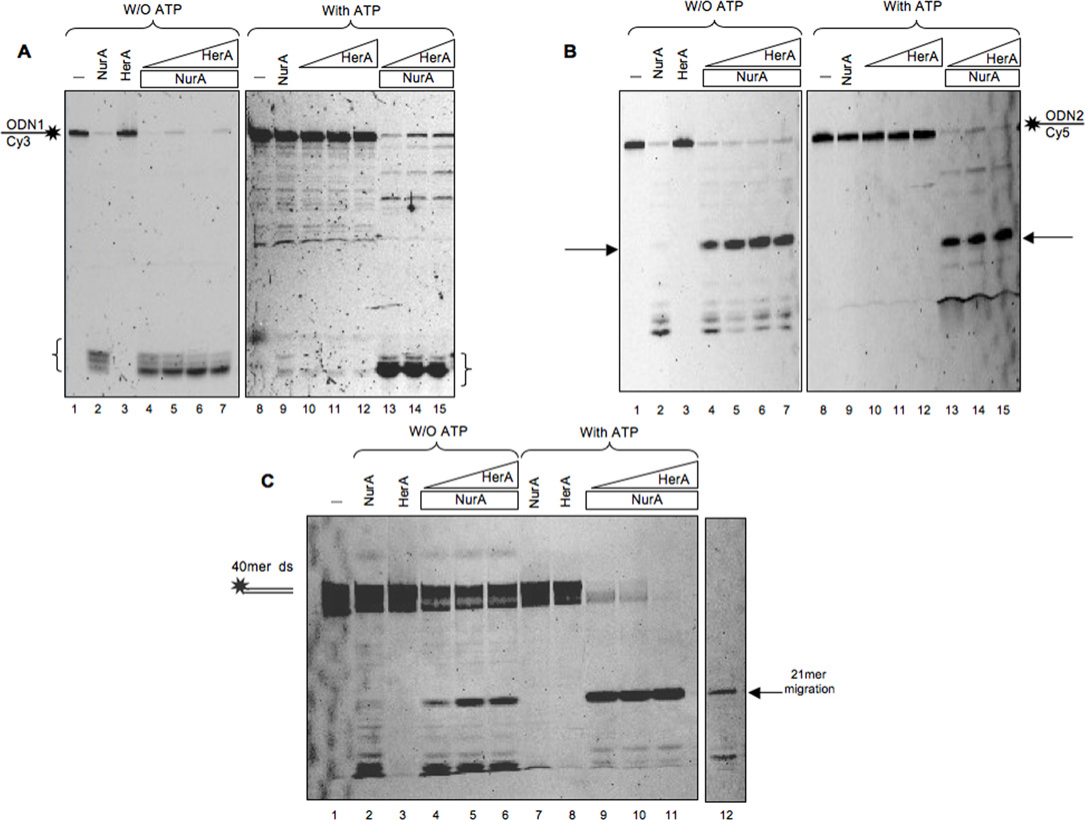
HerA modulates NurA nuclease activity on linear single-stranded DNA. NurA/HerA complex was analyzed for its nuclease activity on single-stranded DNA in the absence or in the presence of 5 mM ATP with ODN1 (A) or ODN2 (B) substrates. The proteins were incubated either separately in the absence of ATP (A and B, lanes 2 and 3) or mixed together using increasing amounts of HerA (from 3 to 18 pmoles) and 18 pmoles of NurA (A and B, lanes 4–7). In the presence of ATP, 7.5 pmoles of NurA and increasing amounts of HerA (from 5 to 12 pmoles) were analyzed either separately (A and B lanes 9–12) or mixed (A and B, lanes 13–15). The main products are indicated by curly braces for ODN1 and arrows for ODN2. Lane 1 refers to a control lane in which no protein has been added. (C) NurA/HerA complex nuclease activity on double stranded DNA. ODN1 has been previously annealed with its complementary strand in order to obtain a linear double-stranded DNA molecule (see Material and Methods and Table 1) 19 pmoles of NurA and 12 pmoles of HerA were analyzed separately without or with 5 mM ATP (lanes 2–3 and 7–8, respectively). Then the same amount of NurA was analyzed in the presence of increasing amounts of HerA (from 3 to 12 pmoles) in the absence or in the presence of ATP (lanes 4–6 and 9–11, respectively). Lane 1 refers to a control in which no protein was added in the assay. Lanes 12 refers to a 21 mer Cy5 used as marker.

In order to compare the pattern of degradation of NurA alone and in the presence of HerA, the products of reaction with the ODN1 and ODN2 substrates were analysed on 15% polyacrylamide gels. As shown in Fig 7, with the ODN1 substrate in the absence of ATP, 18 pmoles of NurA produced three main products (Fig 7A, lane 2). When HerA was added, the same three products were seen but, in this case, the lowest was predominant (Fig 7A, lanes 4–7). As observed with the other substrates, in the presence of ATP NurA showed poor nuclease activity, which was efficiently stimulated by HerA and led to accumulation of the same product (Fig 7A, compare lane 9 with lanes 13–15). The same experiments were performed using ODN2 and, again, the degradation pattern observed in the presence of HerA was different if compared to the one obtained with NurA alone (Fig 7B, compare lane 2 with lanes 4–7); moreover, HerA relieved ATP inhibition of NurA nuclease activtiy (Fig 7B, compare lane 9 with lanes 13–15). In particular, when HerA was present, a new product (indicated by the arrows) with a different migration was observed (Fig 7B). According to Henneke *et al*. [39] this product corresponds to a 4 nucleotides Cy5-labelled molecule since it migrates as a 21-mer substrate (indicated by the arrow in Fig 7C, lane 12). The experiments were also conducted on a double-stranded DNA, in which ODN2 was previously annealed to its complementary strand. As shown in Fig 7C, the pattern observed was comparable to the one obtained for the single stranded ODN2 (Fig 7B). Moreover, comparing the effect of HerA on NurA in the absence (lanes 4–6) or in the presence (lanes 9–11) of ATP, once again, we observed a stronger stimulation when ATP was present.

To further determine whether the activity of NurA/HerA complex was dependent on the substrate, we performed the same experiment on various DNA structures obtained using the 70leadCy5 oligonucleotide previously annealed to different complementary strands (Table 1 and Material and Methods) in order to obtain fork, emi-fork, bubble or double-stranded structures. The degradation pattern was superimposable for all the structures used indicating that the substrate structure does not influence the complex nuclease specificity (data not shown).

**Fig 8:**
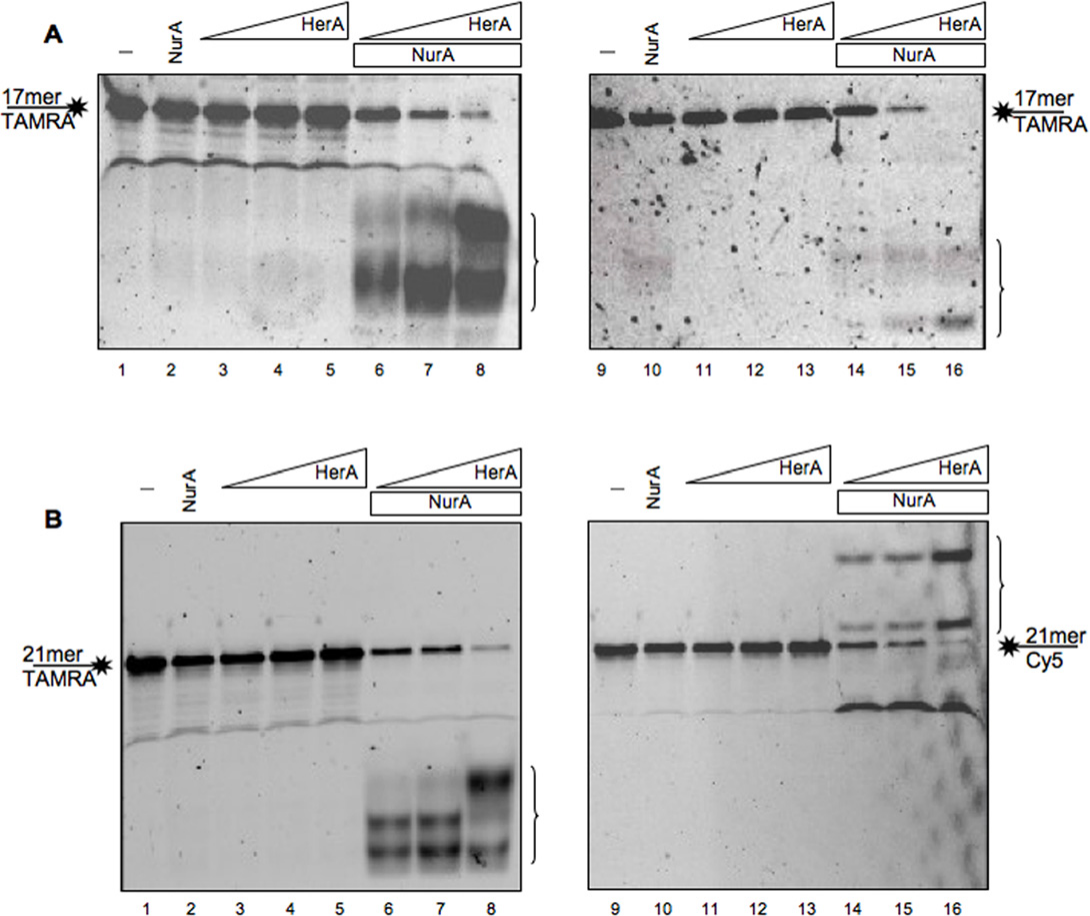
NurA/HerA complex activity on shorter linear single-stranded DNA molecules. (A) 7.5 pmoles of NurA (lanes 2 and 10) or increasing amounts of HerA, from 5 to 12 pmoles (lanes 3–5 and 11–13) have been analyzed separately for their nuclease activity using a linear 17 mer labeled with TAMRA at its 3’- (left) or 5’- (right) ends. The two proteins were then mixed together to analyze the complex activity on these substrates (lanes 6–8 and 14–16). (B) The same experiment was performed using a 21mer single-stranded oligonucleotide labeled at its 3’-end with TAMRA (left) or with Cy5 at its 5’-end (right). 7.5 pmoles of NurA alone (lanes 2 and 10) or increasing amounts of HerA, from 5 to 12 pmoles (lanes 3–5 and 11–13) were analyzed separately. Successively the two proteins were mixed together (lanes 6–8 and 14–16) to analyze the complex nuclease activity. Lanes 1 and 9 are controls in which the only substrate has been run to observe its migration.

In order to define the length of the product released by NurA/HerA complex we used shorter oligonucleotides (Table 1) in the presence of a fixed amount of NurA and increasing concentrations of HerA. As shown in Fig 8A, with a 17 mer substrate the complex produces two main products, while the bands released with a 21 mer substrate appear to be three (Fig 8B). The same number of products was released both if the substrate was labelled on its 3’- or 5’- end. Taken together, these data suggest that the NurA/HerA complex produces fragments of about 6 nucleotides, in agreement with data showed for the *Pf*NurA [36].

## Discussion

In all organisms, initiation of homologous recombination requires the processing of DNA ends in 3’ overhangs, which are required for recombinase, loading and subsequent strand invasion. The process requires many interactive and regulated proteins. This process is highly studied in bacteria and eukarya but is poorly understood in archaea [40].

Our study is focused on the analysis of the mechanism of DNA end-resection in *S*. *solfataricus* and in particular we investigated on NurA nuclease activity alone and in complex with HerA. The genes encoding for these two enzymes are peculiar of thermophilic archaea and are conserved in an operon that includes *mre11* and *rad50* genes. However, the NurA and HerA homologous proteins are present in a few bacteria, such as *Bacillus halodurans*, *Clostridium thermocellum*, *Aquifex aeolicus*, and *D*. *radiodurans* [26]. A variety of genetic and biochemical studies revealed that dedicated and functionally conserved helicase and nuclease activities act in concert at sites of DSB damage; whereas all studies clearly showed a physical and functional interaction of HerA and NurA, contradictory results have been reported on the enzymatic activities of NurA and HerA proteins from different species, their substrate specificity and their mutual dependence [25, 30–37]. Whereas NurA from *S*. *acidocaldarius* was reported to display both ss-endonuclease and 5′→3′ exonuclease activity on ss- and ds-DNA [32], Blackwood *et al*. [30] were unable to detect nuclease activity by the highly similar NurA from *S*. *solfataricus* in the absence of HerA. Moreover, NurA from *P*. *furiosus* was reported to have a weak Mn^2+^ dependent 5′→3′ exonuclease activity, but no nicking activity [25,36]; in contrast, *D*. *radiodurans* NurA showed HerA-independent nicking endonuclease activity against closed circular DNA molecules [37]. Our data were in agreement with some of the above results. We demonstrated that, under our experimental conditions, NurA from *S*. *solfataricus* possesses a strong endonuclease, as well as exonuclease activity on both 5′ and 3′ ends of substrates even in the absence of HerA. We were able to show that the exonuclease, but not endonuclease activity, is stimulated by HerA, suggesting that NurA follows different digestion models in the presence or absence of HerA, (as also reported for the *D*. *radiodurans* protein [37]), in particular we propose that it stimulates the end-resection of six nucleotides of the linear DNA substrate. Moreover, since a stimulation of NurA nuclease activity was observed also on linear double-stranded DNA, an attractive hypothesis is that HerA needs a free-end to load onto DNA and to induce a partial local unwinding that promotes the exposure of a single strand region which becomes target for NurA nuclease activity. In addition, the NurA exonuclease, but not the nicking activity, depends on the D58 conserved residue, suggesting the possible presence of two distinct catalytic sites. Moreover we confirmed a stable physical interaction between NurA and HerA that is in agreement with what has been previously observed with the *P*. *furiosus* and *S*. *solfataricus* enzymes [25,30]. We found that NurA nuclease activity is strongly inhibited by ATP, likely by chelation of divalent ions needed for the reaction. Interestingly, ATP was also reported to inhibit the nuclease activity of the *D*. *radiodurans* NurA protein even in the presence of HerA [37]; in our case, we found that ATP inhibition is not seen in the presence of HerA. Indeed, in agreement with previous reports, we found that HerA stimulates NurA nuclease activity, even in conditions of low NurA activity, such as when Mg^2+^ instead of Mn^2+^ is present. We suggest that the presence of HerA may overcome the inhibition of NurA nuclease activity by ATP due to binding or hydrolysis of the nucleotide, or because the NurA conformation in the HerA-NurA complex is no longer sensitive to such inhibition.

The reasons for the reported differences among the various NurA-HerA proteins are not clear, and may depend on purification protocols and/or experimental assay conditions. Our study contributes further insight in this complex picture, suggesting that NurA may function either alone or in combination with HerA, possibly depending on the physiological conditions: in the presence of appropriate Mn^2+^ concentrations and in the absence of ATP, the enzyme might work on its own; however, in the presence of ATP and/or in the presence of Mg^2+^ ions, NurA activity might require HerA. The inhibition by ATP might thus be a means to restrict the nuclease activity and render it dependent on HerA activity.

## Supporting Information

S1 Fig Homogeneously purified recombinant HerA and NurA. An aliquot (30 l) of HerA (Lane 1, 56 kDa as monomer) or NurA (lane 2, 39 kDa as monomer) were analyzed by SDS-PAGE and Comassie blue staining after last purification step (Heparin affinity column). Lane 3 refers to Molecular Weight markers.
(PDF)

S2 Fig Gel filtration analysis of the interaction between HerA and NurA by a Superdex™ 200 10/300 GL column. A total of 800 μg HerA, 200 μg NurA separately (A and B respectively) or mixed (C) were incubated at 60°C for 20 min before gel filtration. The sample fractions were analyzed by SDS-PAGE. The proteins turned out to be dimer (NurA) and hexamer (HerA) in solution. The elution peaks of the complexes are indicated by arrows at the bottom. The mixed proteins co-eluted earlier than the two proteins run separately, indicating that they form a stable complex
(PDF)

S3 Fig D58A was homogeneously purified. D58A was analyzed by SDS-PAGE after the last step of purification (Heparin affinity column); in order to analyze its homogeneity, three different quantities of recombinant D58A (5, 10 and 20 μl, lanes 2, 3 and 4, respectively) were loaded and visualized by Comassie blue staining.
(PDF)

S4 Fig NurA nuclease activity prefers Mn^2+^ cations. Nuclease assay on ODN1 was performed with NurA (from 4 to 7.5 pmoles of dimer) and 5 mM Mn^2+^ or Mg^2+^ (lanes 2–4 or 6–8, respectively). The same experiment was conducted on ODN2 using the same amount of NurA and cations (lanes 10–12 for Mn^2+^ and 14–16 for Mg^2+^). As clearly shown, NurA prefers Mn^2+^ on both substrates even if, when the substrate used was ODN1, a faint signal can be observed even in the presence of Mg^2+^ (lanes 7–8). Lanes 1, 4, 9 and 13 are controls in which no protein was added in the mixture assay.
(PDF)

S5 Fig HerA is able to modulate NurA nuclease activity even in the presence of Mg^2+^. HerA effect on NurA nuclease activity on ODN1 and ODN2 was analyzed using 12 pmoles of HerA alone (lanes 2 and 10, respectively) and in the presence of increasing amounts of NurA (1, 2 and 4 pmoles; lanes 6–8 for ODN1 and 14–16 for ODN2). The same concentrations of NurA were analyzed in the absence of HerA as control (lanes 3–5 for ODN1 and 11–13 for ODN2). Lanes 1 and 9 are controls experiments in which no protein was added. On both substrates we see some degradation products only when HerA was present in the reaction, indicating that HerA is able to modulate NurA nuclease activity even in the presence of Mg^2+^, even if not with the same efficiency observed with Mn^2+^.
(PDF)
