# Supplementary material for: NurA Is Endowed with Endo- and Exonuclease Activities that Are Modulated by HerA: New Insight into Their Role in DNA-End Processing
Source: PLoS One. 2015 Nov 11;10(11):e0142345. doi: 10.1371/journal.pone.0142345 (PMC4641729; doi:10.1371/journal.pone.0142345)
Supplement: S1 Fig — An aliquot (30 l) of HerA (Lane 1, 56 kDa as monomer) or NurA (lane 2, 39 kDa as monomer) were analyzed by SDS-PAGE and Comassie blue staining after last purification step (Heparin affinity column). Lane 3 refers to Molecular Weight markers. (PDF) [file pone.0142345.s001.pdf]

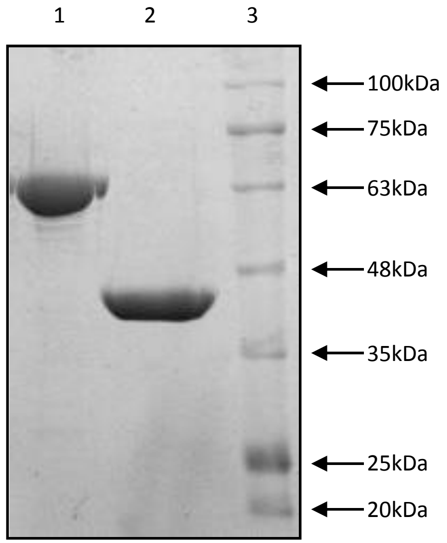

Supplementary Figure S1: Homogeneously purified recombinant HerA and NurA. An aliquot (30 μl) of HerA (Lane 1, 56 kDa as monomer) or NurA (lane 2, 39 kDa as monomer) were analyzed by SDS-PAGE and Comassie blue staining after last purification step (Heparin affinity column). Lane 3 refers to Molecular Weight markers.
