# Supplementary material for: NurA Is Endowed with Endo- and Exonuclease Activities that Are Modulated by HerA: New Insight into Their Role in DNA-End Processing
Source: PLoS One. 2015 Nov 11;10(11):e0142345. doi: 10.1371/journal.pone.0142345 (PMC4641729; doi:10.1371/journal.pone.0142345)
Supplement: S2 Fig — A total of 800 μg HerA, 200 μg NurA separately (A and B respectively) or mixed (C) were incubated at 60°C for 20 min before gel filtration. The sample fractions were analyzed by SDS-PAGE. The proteins turned out to be dimer (NurA) and hexamer (HerA) in solution. The elution peaks of the complexes are indicated by arrows at the bottom. The mixed proteins co-eluted earlier than the two proteins run separately, indicating that they form a stable complex (PDF) [file pone.0142345.s002.pdf]

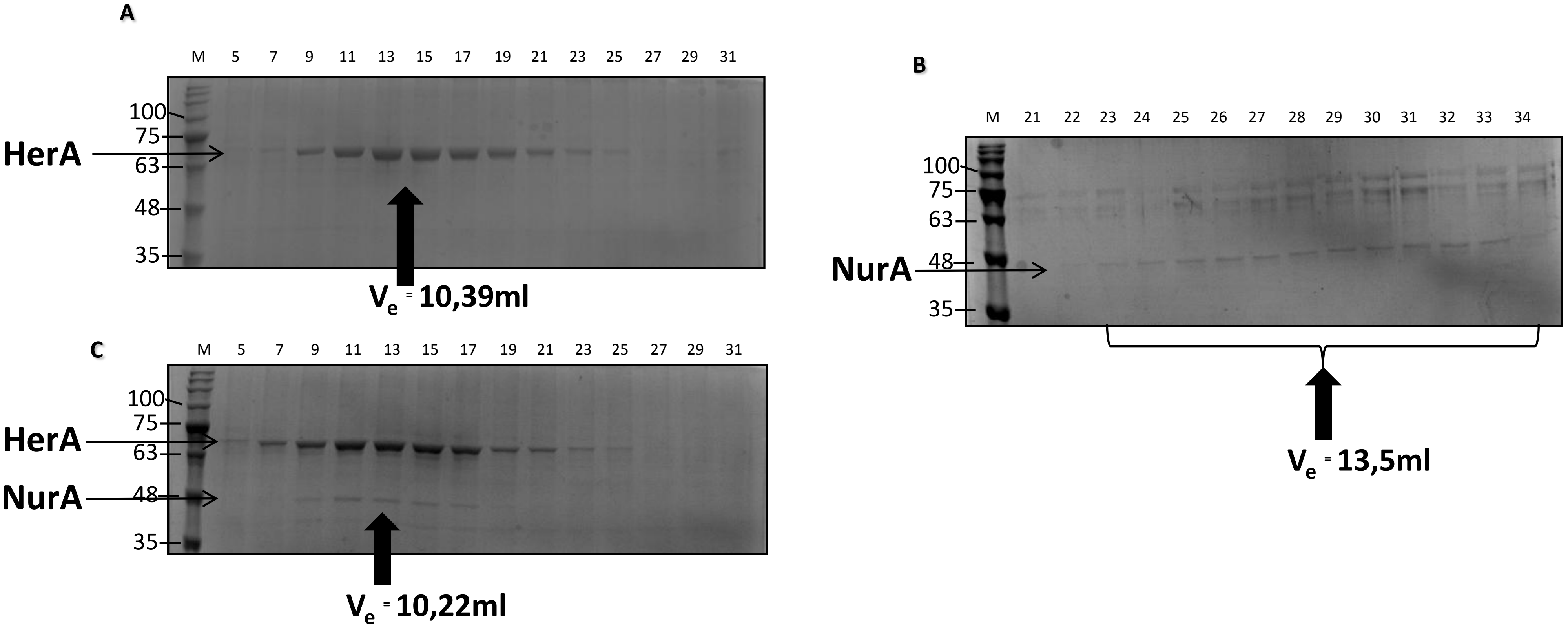

Supplementary Figure S2: Gel filtration analysis of the interaction between HerA and NurA by a Superdex™ 200 10/300 GL column. A total of 800 µg HerA, 200 µg NurA separately (A and B respectively) or mixed (C) were incubated at 60°C for 20 min before gel filtration. The sample fractions were analyzed by SDS-PAGE. The proteins turned out to be dimer (NurA) and hexamer (HerA) in solution. The elution peaks of the complexes are indicated by arrows at the bottom. The mixed proteins co-eluted earlier than the two proteins run separately, indicating that they form a stable complex
