# Supplementary material for: NurA Is Endowed with Endo- and Exonuclease Activities that Are Modulated by HerA: New Insight into Their Role in DNA-End Processing
Source: PLoS One. 2015 Nov 11;10(11):e0142345. doi: 10.1371/journal.pone.0142345 (PMC4641729; doi:10.1371/journal.pone.0142345)
Supplement: S3 Fig — D58A was analyzed by SDS-PAGE after the last step of purification (Heparin affinity column); in order to analyze its homogeneity, three different quantities of recombinant D58A (5, 10 and 20 μl, lanes 2, 3 and 4, respectively) were loaded and visualized by Comassie blue staining. (PDF) [file pone.0142345.s003.pdf]

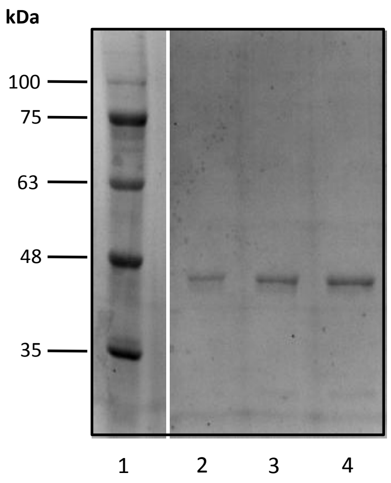

Supplementary figure S3: D58A was homogeneously purified. D58A was analyzed by SDS-PAGE after the last step of purification (Heparin affinity column); in order to analyze its homogeneity, three different quantities of recombinant D58A (5, 10 and 20 µl, lanes 2, 3 and 4, respectively) were loaded and visualized by Comassie blue staining.
