# Supplementary material for: NurA Is Endowed with Endo- and Exonuclease Activities that Are Modulated by HerA: New Insight into Their Role in DNA-End Processing
Source: PLoS One. 2015 Nov 11;10(11):e0142345. doi: 10.1371/journal.pone.0142345 (PMC4641729; doi:10.1371/journal.pone.0142345)
Supplement: S4 Fig — Nuclease assay on ODN1 was performed with NurA (from 4 to 7.5 pmoles of dimer) and 5 mM Mn2+ or Mg2+ (lanes 2–4 or 6–8, respectively). The same experiment was conducted on ODN2 using the same amount of NurA and cations (lanes 10–12 for Mn2+ and 14–16 for Mg2+). As clearly shown, NurA prefers Mn2+ on both substrates even if, when the substrate used was ODN1, a faint signal can be observed even in the presence of Mg2+ (lanes 7–8). Lanes 1, 4, 9 and 13 are controls in which no protein was added in the mixture assay. (PDF) [file pone.0142345.s004.pdf]

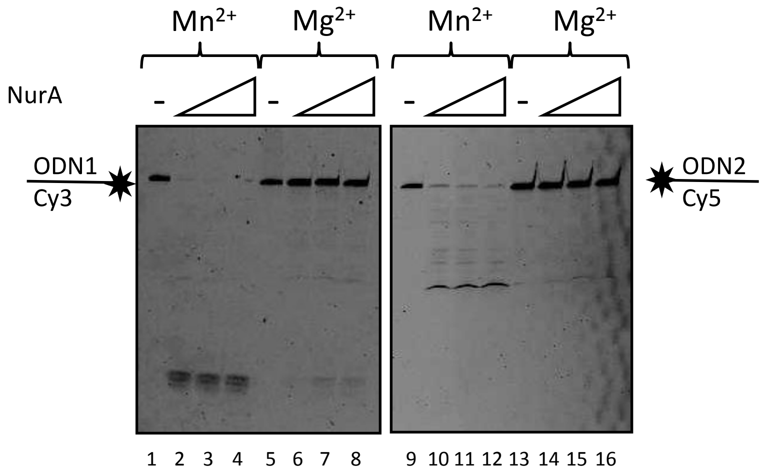


Supplementary Figure S4: NurA nuclease activity prefers $Mn^{2+}$ cations. Nuclease assay on ODN1 was performed with NurA (from 4 to 7.5 pmoles of dimer) and 5 mM $Mn^{2+}$ or $Mg^{2+}$ (lanes 2-4 or 6-8, respectively). The same experiment was conducted on ODN2 using the same amount of NurA and cations (lanes 10-12 for $Mn^{2+}$ and 14-16 for $Mg^{2+}$). As clearly shown, NurA prefers $Mn^{2+}$ on both substrates even if, when the substrate used was ODN1, a faint signal can be observed even in the presence of $Mg^{2+}$ (lanes 7-8). Lanes 1, 4, 9 and 13 are controls in which no protein was added in the mixture assay.
