# Supplementary material for: NurA Is Endowed with Endo- and Exonuclease Activities that Are Modulated by HerA: New Insight into Their Role in DNA-End Processing
Source: PLoS One. 2015 Nov 11;10(11):e0142345. doi: 10.1371/journal.pone.0142345 (PMC4641729; doi:10.1371/journal.pone.0142345)
Supplement: S5 Fig — HerA effect on NurA nuclease activity on ODN1 and ODN2 was analyzed using 12 pmoles of HerA alone (lanes 2 and 10, respectively) and in the presence of increasing amounts of NurA (1, 2 and 4 pmoles; lanes 6–8 for ODN1 and 14–16 for ODN2). The same concentrations of NurA were analyzed in the absence of HerA as control (lanes 3–5 for ODN1 and 11–13 for ODN2). Lanes 1 and 9 are controls experiments in which no protein was added. On both substrates we see some degradation products only when HerA was present in the reaction, indicating that HerA is able to modulate NurA nuclease activity even in the presence of Mg2+, even if not with the same efficiency observed with Mn2+. (PDF) [file pone.0142345.s005.pdf]

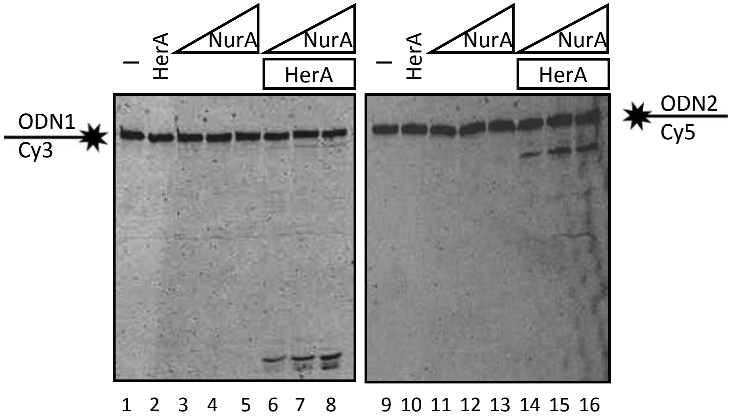

Supplementary figure S5: HerA is able to modulate NurA nuclease activity even in the presence of Mg^{2+}. HerA effect on NurA nuclease activity on ODN1 and ODN2 was analyzed using 12 pmoles of HerA alone (lanes 2 and 10, respectively) and in the presence of increasing amounts of NurA (1, 2 and 4 pmoles; lanes 6-8 for ODN1 and 14-16 for ODN2). The same concentrations of NurA were analyzed in the absence of HerA as control (lanes 3-5 for ODN1 and 11-13 for ODN2). Lanes 1 and 9 are controls experiments in which no protein was added. On both substrates we see some degradation products only when HerA was present in the reaction, indicating that HerA is able to modulate NurA nuclease activity even in the presence of Mg^{2+}, even if not with the same efficiency observed with Mn^{2+}.
